# Comparative anatomy and salt management of *Sonneratia caseolaris* (L.) Engl. (Lythraceae) grown in saltwater and freshwater

**DOI:** 10.7717/peerj.10962

**Published:** 2021-02-25

**Authors:** Sukrit Tatongjai, Ekaphan Kraichak, Prasart Kermanee

**Affiliations:** Department of Botany, Faculty of Science, Kasetsart University, Bangkok, Thailand

**Keywords:** *Sonneratia caseolaris*, Comparative anatomy, Mangrove, Saltwater, Freshwater

## Abstract

*Sonneratia caseolaris* is a pioneer species in mangrove. It can naturally grow in both saltwater and freshwater. The study was aimed at investigating and comparing the anatomical character of the *S. caseolaris* plants growing in different conditions and how they coped with salinity. The anatomical characteristics of roots, stems, petioles and leaf blade were investigated. The plant samples were prepared into permanent slides using a paraffin method, while the wood samples were made into permanent slides using a sliding microtome technique. Tissue clearing of leaf blade and scanning electron microscopic analysis of wood were performed. In addition, sodium chloride content in various organs and tissues was examined. It was found that cable root, stem and leaf blade showed some different anatomical characteristics between the two conditions. Periderm is a prominent tissue in saltwater roots. Tanniferous cells were observed in pneumatophores, petioles, stems and leaf blades of saltwater plants, but not found in pneumatophores and lamina of freshwater plants. Mesophyll thickness was lower in the saltwater condition. The vessel density was significantly higher in the saltwater condition than in the freshwater condition, whereas the vessel diameters in the freshwater condition were significantly higher than those in the saltwater condition. From the results, it can be concluded that root periderm plays an important role in salt exclusion, and the occurrence of tanniferous cells is associated with salt elimination.

## Introduction

Mangrove is a plant community that grows in the interface between land and sea of tropical and sub-tropical latitudes (Kathiresan & Bingham, 2001). The major environmental factors in this habitat are high salinity and tidal fluctuations. Salinity has long been recognized as an important factor that limits mangrove growth, seedling biomass, and productivity (Ball, 2002; Clough & Sim, 1989; Dissanayake et al., 2018; Hasegawa et al., 2000; Hoppe-Speer et al., 2011; Kodikara et al., 2017; Lin & Sternberg, 1992). To deal with the salt stress, different mangrove species use different combinations of the three salt eliminating mechanisms: salt secretion, salt exclusion and salt accumulation (Parida & Jha, 2010). In addition, the mangrove plants also need to adapt for flooding stress from tidal fluctuation, which alters the salinity condition throughout the day. Therefore, mangrove plants are constantly presented with these stresses and must have some adaptations to deal with this challenging environment.

Many changes in some aspects of mangrove morphology and anatomy seem to be the result of plant adaptations to salt stress. For instance, leaves of *Aegialitis* and *Avicennia* have special glands to eliminate excess salts, whereas other species such as *Rhizophora* and *Sonneratia* do not possess these glands (Reef & Lovelock, 2015; Scholander et al., 1962). Roots of mangrove plants exhibit aerenchyma in response to anaerobic conditions. Aerenchyma has large air spaces that enable the rapid transport of gases (e.g., oxygen, carbon dioxide, ethylene, and methane) (Armstrong, 1980; Colmer, 2003; Evans, 2003; Jackson & Armstrong, 1999). Gases can be transported throughout the root leading to increasing metabolic efficiency, despite the low oxygen condition outside the root in the soil (Purnobasuki & Suzuki, 2004). Some genera, such as *Rhizophora*, eliminates salt by an ultrafiltration mechanism occurring at the membranes of root (Scholander et al., 1966; Scholander, 1968), while *Lumnitzera* and *Excoecaria* develop succulent leaves to accumulate water that can dilute salts in the vacuoles (Kathiresan & Bingham, 2001; Tomlinson, 1986). These examples show that the same harsh environment of mangrove forests can lead to different strategies among sympatric mangrove species.

**Figure 1:**
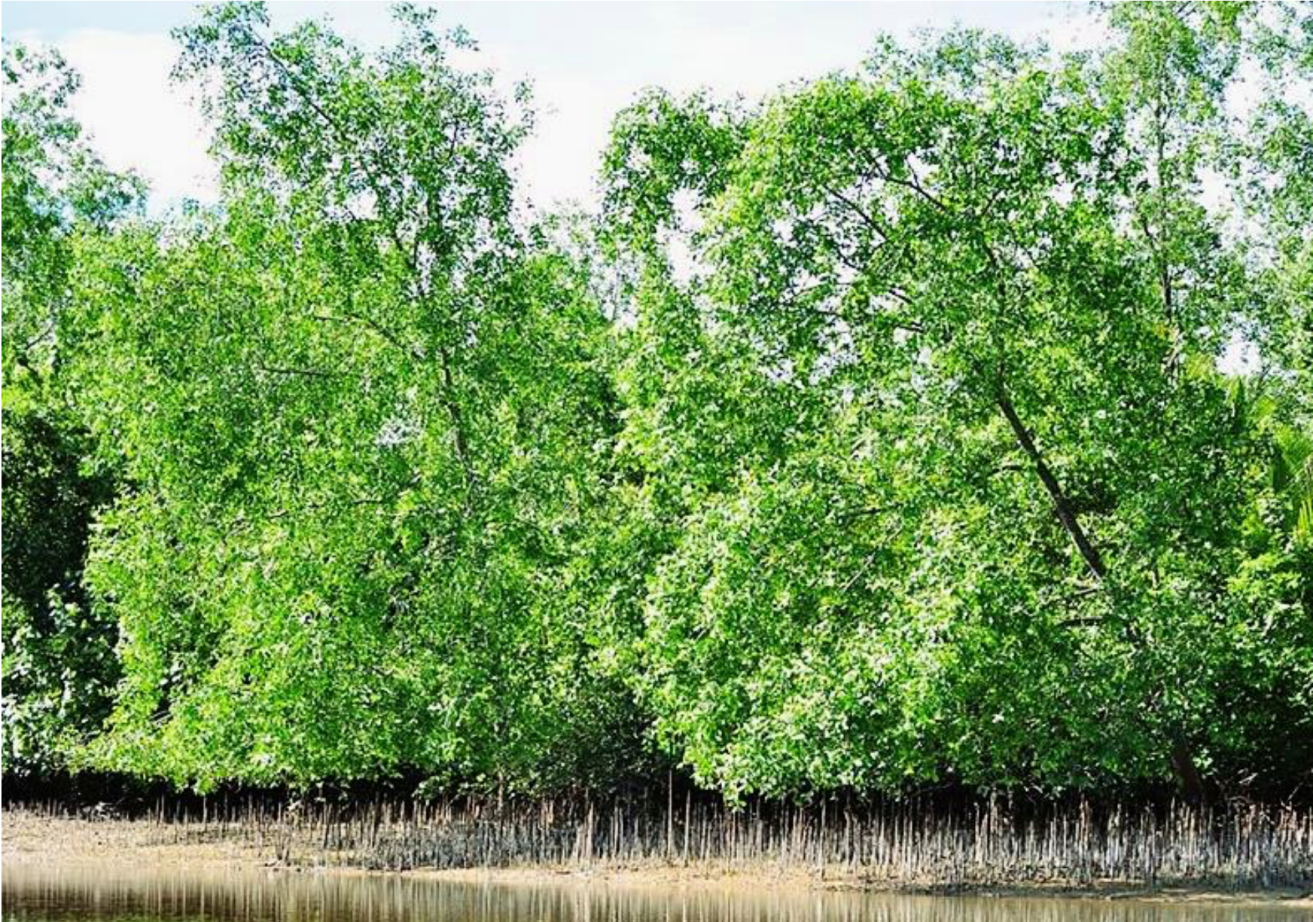
Plant habit of *Sonneratia caseolaris* grown in saltwater.

**Figure 2:**
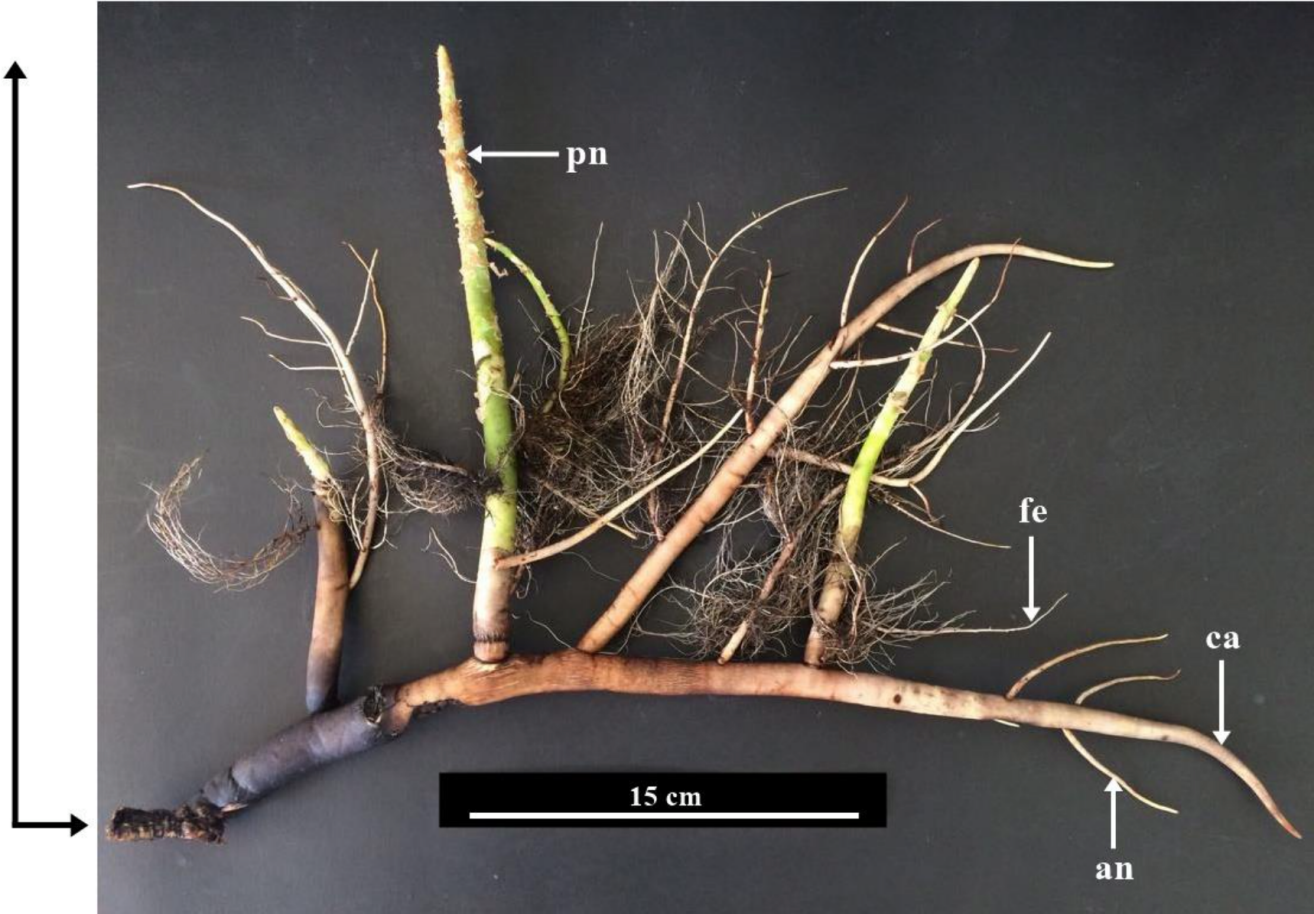
Root system of *Sonneratia caseolaris* grown in saltwater showing the differrent types of root observed. Abbreviations: ca, cable root; pn, pneumatophore; fe, feeding root; an, anchor root.

*Sonneratia caseolaris* (L.) Engl. (Lythraceae) is a pioneer and true mangrove species that is native to the Sundarbans and Chittagong areas of Bangladesh. This species is a medium–large tree, evergreen, with simple leaves usually elliptic to narrowly ovate or obovate (Bunyavejchewin & Buasalee, 2011) (Fig. 1). It is easily recognized by its flowers and fruits. Interestingly, *S. caseolaris* develops a highly specialized root system with multiple root types (Fig. 2). *S. caseolaris* was traditionally used as an astringent and antiseptic, treating piles and sprain poultices (Bandaranayake, 1998). It is also used as an ornamental plant. Stems and woods are used for firewood, building boats, posts of bridges and houses (Wetwitayaklung, Limmatvapirat & Phaechamud, 2013).

Ecologically, *Sonneratia caseolaris* occurs in the upstream estuarine zone in lower saline areas with deep muddy soil, which allows this species to thrive in brackish to freshwater environments. However, detailed comparisons of anatomical structures of these plants in fresh and saltwater has not been conducted to date. Therefore, in this paper, we comparatively investigated anatomical characteristics of *S. caseolaris*, which grows in the habitats with different salinity. The research problems addressed in this study was how the anatomical structures of this plant were correlated with the high salinity environment. It was hypothesized that leaf anatomical characters would be different between the plants growing in different salinity conditions. The plant samples from vegetative organs were sectioned and macerated to examine their internal structures. The sodium chloride contents were also measured in these organs to determine the level of salt accumulation.

## Materials and Methods

### Sampling

Samples of *S. caseolaris* were kindly supplied by Sirinart Rajini Ecosystem Learning Center and authorized by the manager Komson Hongpadharakiree, in Prachuap Kiri Khan Province, next to the Gulf of Thailand (12°23′40.8″N 99°58′52.8″E) in December 2017. The salinity in this mangrove forest in December 2017 was 5,885.00 ± 1.10 ppm. The salinity and water levels of the Gulf of Thailand can vary seasonally. The lowest of salinity is in January, and the maximum is in July with an annual average of about 32,300 ppm (Stansfield & Garrett, 1997). The tidal cycle at the sampling site is diurnal with the highest water level in December to January at 0.2 to 0.3 m from mean sea level (MSL) and the lowest level in August to September at −0.5 to −0.4 m from MSL (Sojisuporn, Morimoto & Yanagi, 2010).

The sampling from a freshwater environment were kindly supplied from the Botanical Garden of Department of Agriculture, Bangkok, Thailand (13°51′15.6″N 100°34′26.1″E). The plant used as an ornamental plant which grown along the channel of fresh water. The salinity of this freshwater environment in December 2017 was 120.20 ± 0.98 ppm. Various parts of the plants were used for the anatomical study, including four types of roots (cable root, pneumatophore, feeding root and anchor root) (Fig. 2), stems, petioles and leaf blades.

### Permanent slide preparation

#### Paraffin method

The plant materials, including the four types of roots (3 cm from the root tip), stems (1 cm from the shoot tip for primary growth, 5 cm from the shoot tip for secondary growth), and petioles with leaf blades (the third leaf pair from shoot) were fixed in 50% FAA (50% ethanol, 5% formalin, and 5% glacial acetic acid) for 24 h, subjected to a vaccum pump for 1 h and then dehydrated with a tertiary butyl alcohol series (50%, 70%, 85%, 95% and 100%, respectively). Dehydrated samples were embedded in a paraplast. Samples were then sectioned at 15–20 µm using a rotary microtome (Leica RM 2165). Sections were affixed on slides and stained with safranin and fast green combination. The permanent mounting of the slides was performed with Permount (Fisher, USA) (Johansen, 1940; Kermanee, 2008). Twenty permanent slides were prepared for each organ.

### Sliding microtome method

Wood samples were trimmed into a 1 × 1 × 3 cm piece and sectioned with a sliding microtome at 20–30 µm. Sections were stained with safranin (1%) and dehydrated with an ethanol series (30%, 50%, 70%, 95% and 100%, respectively), followed by immersion in a mixture of absolute ethanol and xylene (1:1). The samples were left in xylene for at least 3 h prior to mounting with Permount (Fisher, USA) (Kermanee, 2008).

### Tissue maceration technique

Wood samples were chipped into small pieces, then boiled in a mixture of acetic acid (conc.) and hydrogen peroxide (1:1). The macerated cells were washed, stained with safranin, dehydrated with ethanol series and mounted with Permount (Fisher, USA) (Kermanee, 2008).

### Tissue clearing

Leaf blades were boiled in 10% potassium hydroxide for 3 min. Samples were washed with water and then immersed in Clorox^®^ for 30 min, followed by 50% chloral hydrate for 1 h. Samples were dehydrated with ethanol series and leaved in absolute ethanol and xylene (1:1) for 1 h. Finally, Samples were immersed in xylene prior to mounting with Permount (Fisher, USA) (Kermanee, 2015).

### Scanning electron microscopic technique

Wood samples were cut in to 0.5 × 0.5 × 3 cm^3^ block. The blocks were sectioned with a sliding microtome at 120 µm thickness. Sections were dried in a hot air oven (80° C) for 48 h and then attached on a stub with a double-sided carbon tape. Samples were coated with gold particles using a sputter coater machine (SC7620; Quorum, England) and observed under a scanning electron microscope (JSM 5600 LV; JEOL, Tokyo, Japan).

### Sodium chloride measurement

Sodium chloride concentrations in surrounding waters from both conditions were measured using a water analysis meter (PCD 650, Eutech, USA) with five replications for each of the conditions.

Various tissues (epidermis, cortex, bark, periderm and stele) of root, stem and leaf blade were isolated. The samples were dried in a hot air oven at 60 °C for 1 week. The materials were ground into powder. Five grams of dry weight samples were dissolved in 50 ml deionized water. Sodium chloride concentrations were measured using a water analysis meter (PCD 650, Eutech, USA) with five replicates for each sample.

### Data collection and analysis

Leaf blade thickness, vessel density per square millimeter and vessel diameter were measured from the photographs taken from a Zeiss microscope with Axioskop II Plus software. Thirty measurements were performed for each sample. The *t*-test was performed to determine the difference between the samples from fresh and saltwater, using the R software (R. 3.5.2) (R Core Development Team, 2018).

## Results

*Sonneratia caseolaris* has four types of roots including cable root, pneumatophore, feeding root, and anchor root (Figs. 1–2). The anatomical features of each type of root developing in freshwater and saltwater are described in the following sections.

### Cable root

**Figure 3:**
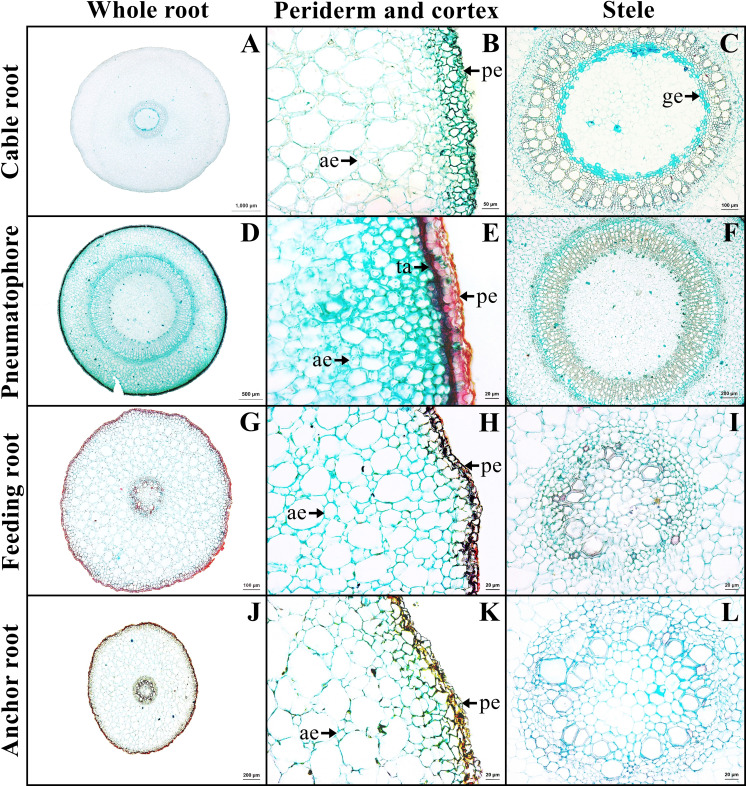
A transverse sections of four types of *Sonneratia caseolaris* root growing in saltwater. (A–C) Cable root. (D–E) Pneumatophore. (G–I) Feeding root. (J–L) Anchor root. Abbreviations: ae, aerenchyma; ge, gelatinous fiber; pe, periderm; ta, tanniferous cell.

The outer layer is the periderm, which includes phellem, phellogen, and phelloderm. The cortex contains aerenchyma with large air spaces. Cortical cells comprised two types: round cells and arm-shaped cells with three to four protruding arms. Druse crystals and sclereids were found in the cortex. The endodermis is the innermost layer of the cortex of mostly rectangular cells. Casparian strips were not observed in endodermis. The stele contains a layer of pericycle with rectangular cells, rings of phloem and xylem, and pith with parenchyma. Gelatinous fibers were observed in the xylem of saltwater plant (Figs. 3A–3C, Supplemental Information 2).

### Pneumatophore

The outer layer is the periderm. Cortex consists of two types of parenchyma: the round parenchyma and aerenchyma. The round parenchyma is adjacent to the periderm and stele. In saltwater plants, some round parenchyma cells store tannin. The second type is aerenchyma with large air spaces. Druse crystals and sclereids were observed in the cortex. A distinct endodermis marked the innermost layer of the cortex. The stele contains a layer of pericycle, rings of phloem and xylem, and pith comprised round parenchyma (Figs. 3E–3F).

### Feeding root

The outer layer is periderm. Cortex consists of two types. The round-elliptic parenchyma cells are closer to periderm and stele. The aerenchyma are formed as a large lacunate cortex. Druse crystals were observed in the arm cells of the cortex, as well as endodermis with Casparian strips. Vascular bundles are the radial type. Pith is the innermost layer of tissue, which consists of only parenchyma cells (Figs. 3G–3I).

### Anchor root

Anchor root has the same structure as feeding root (Figs. 3J–3L).

### Primary and secondary growth stem

The stem in the primary growth stage has a single layer of epidermal cells. Cortex consists of collenchyma and parenchyma. Tanniferous cells were found in the cortex of saltwater plants.

**Figure 4:**
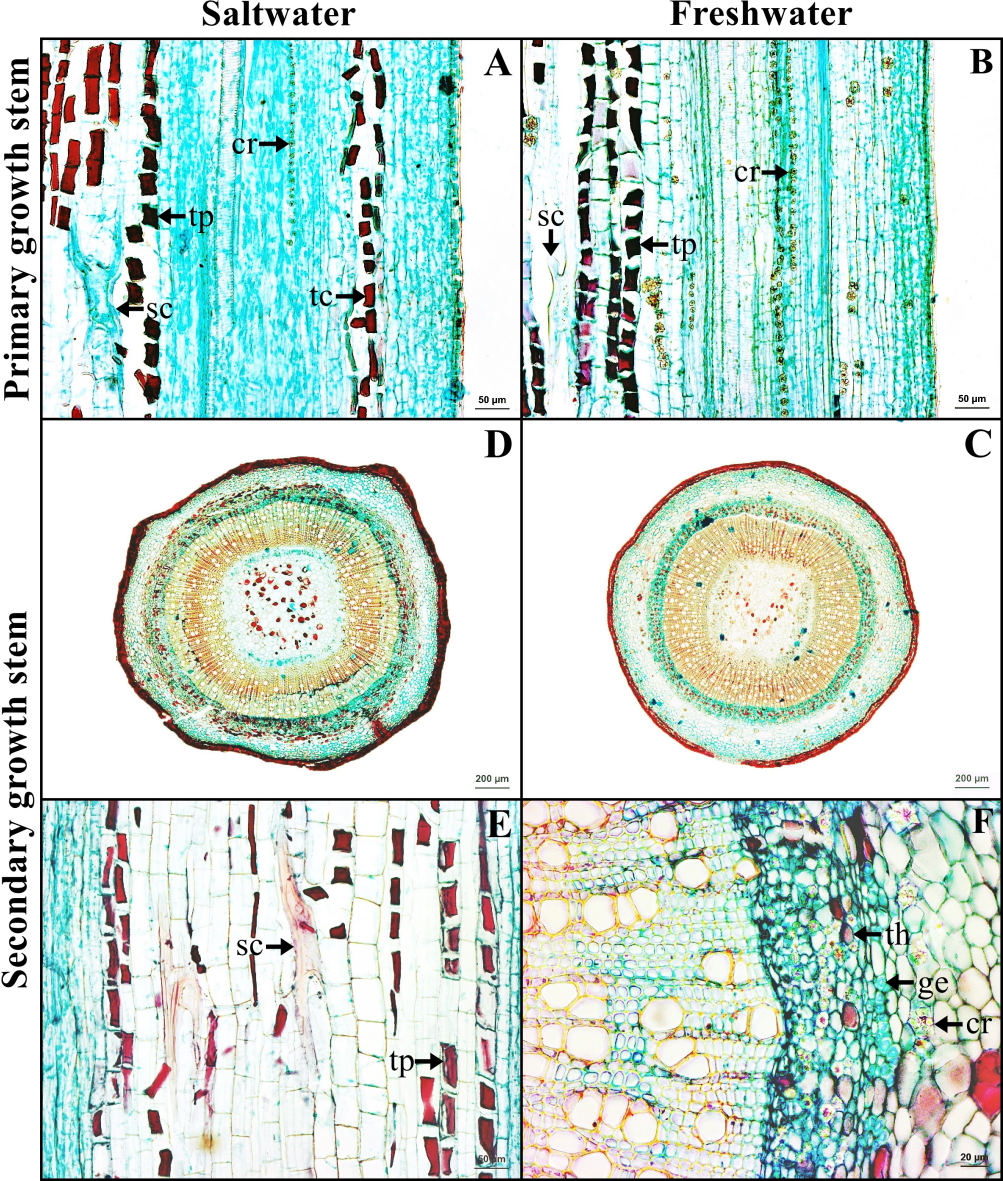
A transverse and longitudinal sections of stem of *Sonneratia caseolaris*. (A–B) Primary growth stem from saltwater and freshwater. (C–D) Secondary growth stem from saltwater and freshwater. (E) Sclereid and tanniferous cell in pith. (F) Gelatinous fiber and tanniferous cell in phloem and druse crystal surround vascular tissue. Abbreviations: cr, druse crystal; ge, gelatinous fiber; sc, sclereid; tc, tanniferous cell in cortex; th, tanniferous cell in phloem; tp, tanniferous cell in pith.

The stem in the secondary growth is composed of periderm, cortex, vascular tissue and pith. Tanniferous cells, druse crystals and sclereids were observed in the cortex of plants from both habitats (Figs. 4A–4B). Gelatinous fibers were observed in the phloem. Pith is the innermost layer of the tissue, comprised of parenchyma and sclereids. Druse crystals and tanniferous cells were also found in phloem and pith (Figs. 4C–4F).

### Petiole

**Figure 5:**
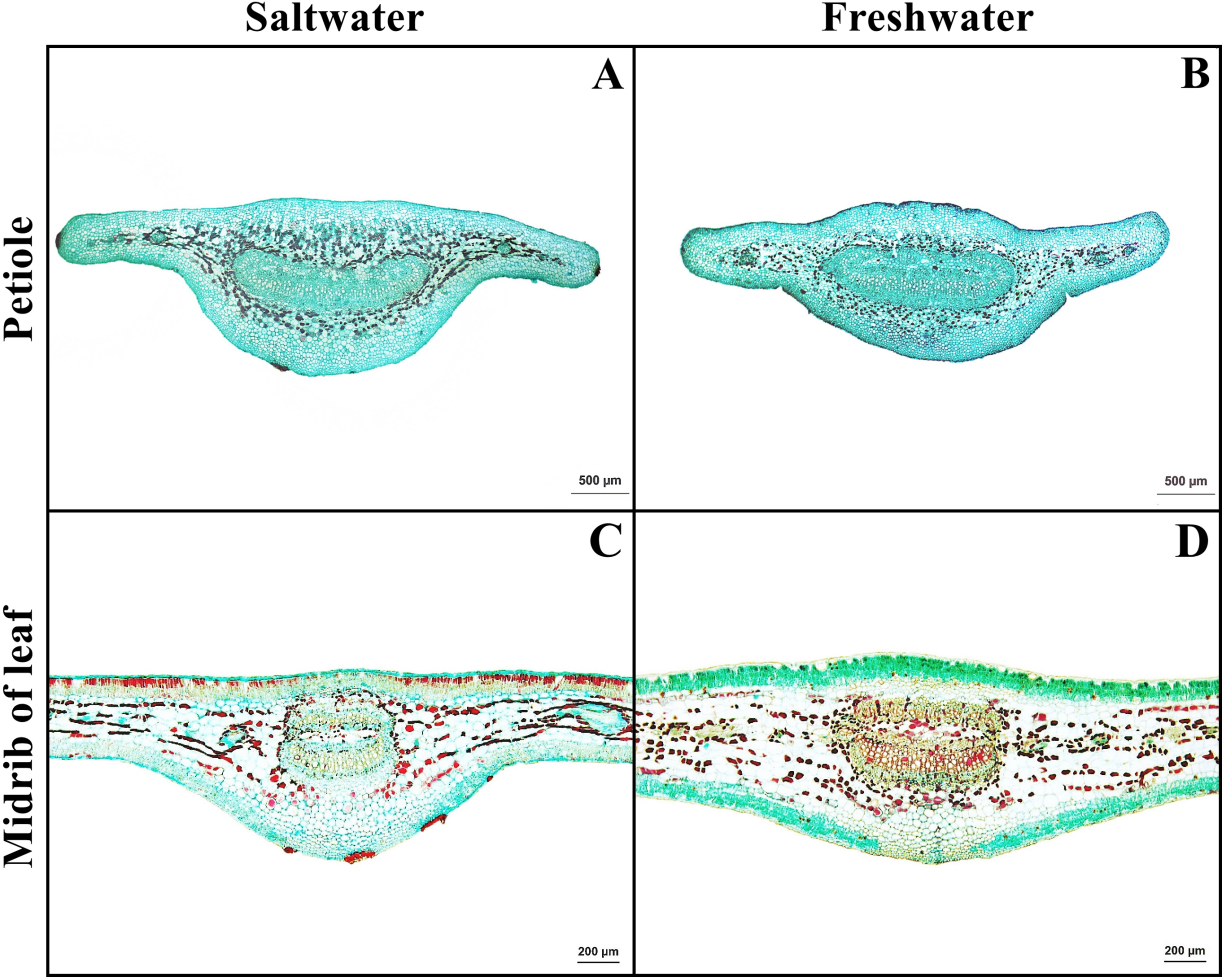
A transverse sections of petiole and leaf blade of *Sonneratia caseolaris*. (A) Petiole from saltwater. (B) Petiole from freshwater. (C) Midrib of leaf blade from saltwater. (D) Midrib of leaf blade from freshwater.

Petiole has one layer of epidermal cells. Cortex consists of four types of cells, collenchyma, parenchyma, sclerenchyma, and tanniferous cells. Druse crystals were observed in the parenchyma in both habitats (Figs. 5A–5B).

### Leaf blade

**Figure 6:**
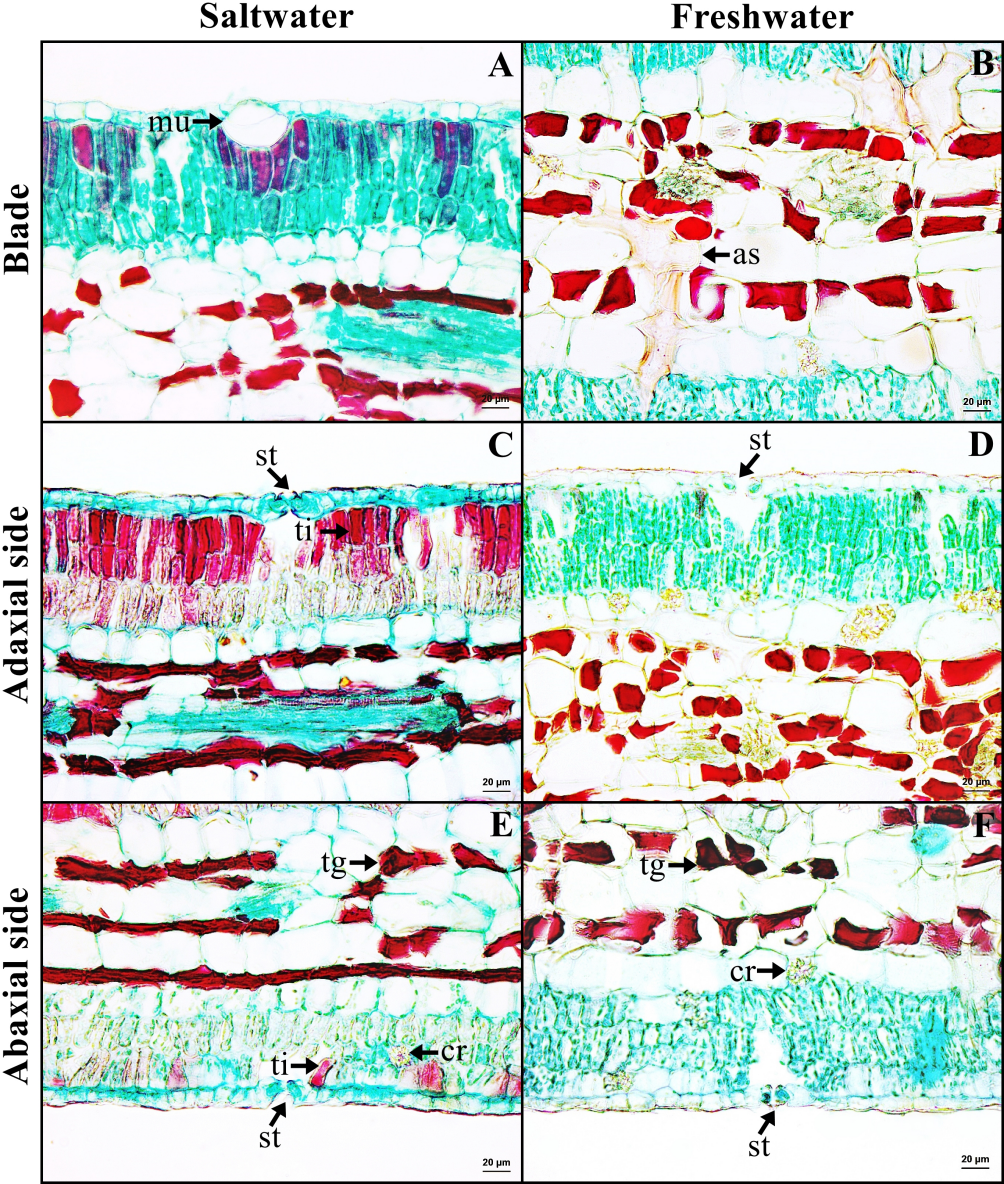
A transverse sections of leaf blade of *Sonneratia caseolaris*. (A) Mcilaginous cavity on adaxial side from saltwater. (B) Sclereid in spongy parenchyma from freshwater. (C–D) Typical stomata and palisade parenchyma on adaxial side of saltwater and freshwater. (E–F) Typical stomata and palisade parenchyma on abaxial side of saltwater and freshwater. Abbreviations: as, astrosclereid; cr, druse crystal; mu, mucilaginous cavity; st, typical stomata; tg, tanniferous cell in spongy parenchyma; ti, tanniferous cell in palisade parenchyma.

Leaf blade is the unifacial type. The leaf tissue is composed of adaxial and abaxial palisade parenchyma. Mucilaginous cavities were found on adaxial side. Typical stoma and staurocytic stoma were observed on both sides of epidermis. The mesophyll layer is composed of two types of modified parenchyma: palisade parenchyma on the both sides and spongy parenchyma in the middle layer. Tannins are commonly observed in ground tissue but absent in palisade parenchyma of freshwater leaf. Astrosclereids are present in the mesophylls of leaf blade. The vascular bundle is bicollateral type. Druse crystals were observed in phloem and leaf mesophyll in both habitats (Figs. 5C–5D, Fig. 6). The average total thickness of freshwater leaves were significantly higher than that saltwater samples (*t*-test, *P* < 0.001, Table 1).

### Wood

**Figure 7:**
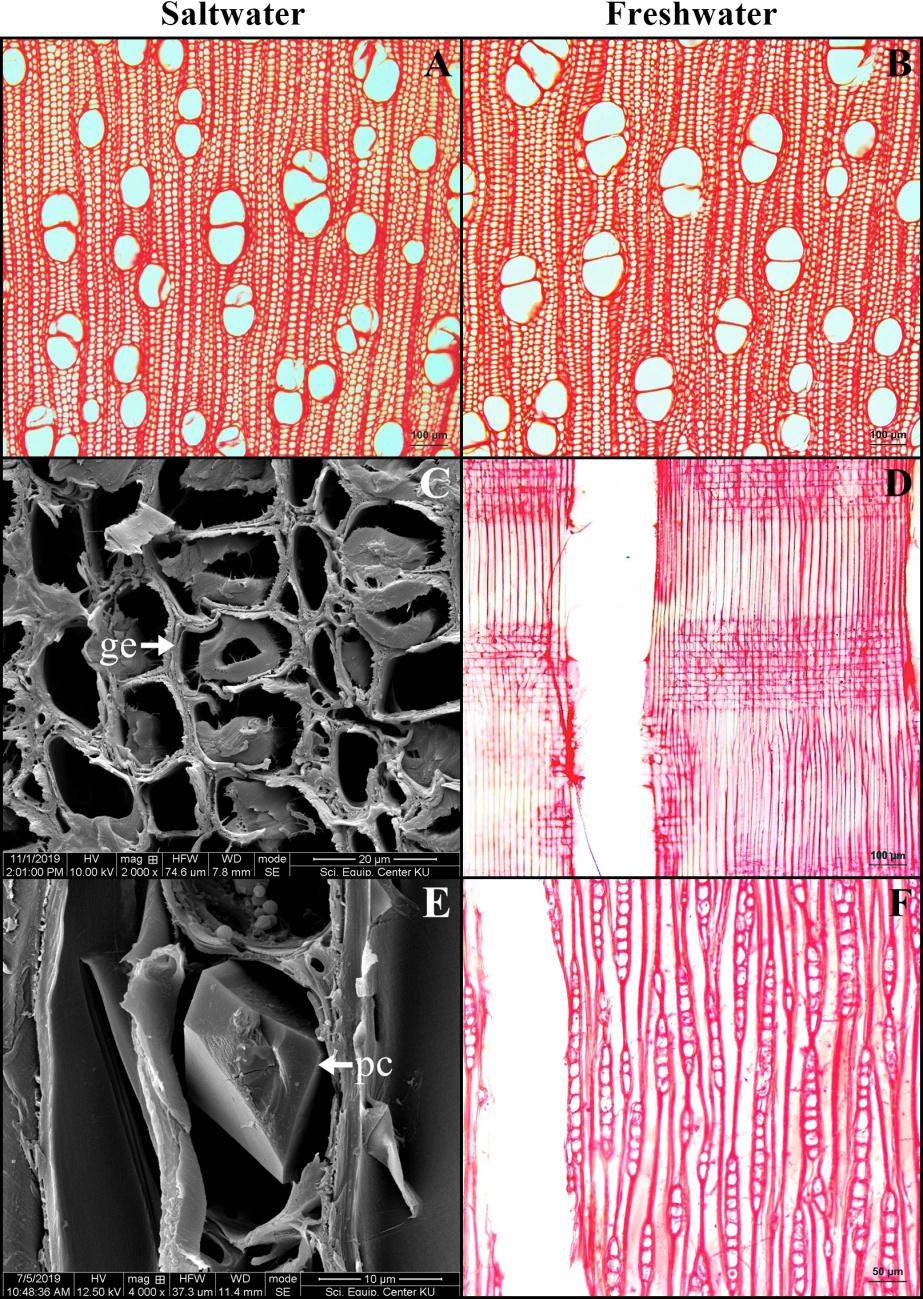
A transverse sections and longitudinal sections of wood of *Sonneratia caseolaris*. (A–B) Transverse sections from saltwater and freshwater. (C) Gelatinous fiber under SEM. (D) Radial section from freshwater. (E) Prismatic crystal in ray cell under SEM. (F) Tangential section from freshwater Abbreviations: ge, gelatinous fiber; pc, prismatic crystal.

The wood is diffuse porous. Pore arrangement is solitary, radial multiple and pore clusters with circular to oval shaped. Vessels have simple perforations. The density of vessel was significantly higher in the saltwater samples than that of the freshwater (26.37 ± 4.37 pores/mm^2^ in saltwater and 23.33 ± 2.11 pores/mm^2^ in freshwater wood, *t*-test, *P* = 0.002). The average of vessel diameter is 79.97 ± 10.51 µm in saltwater, which was significantly smaller than that in freshwater at 84.39 ± 12.46 µm (*t*-test, *P* = 0.049). Woods from both conditions contain libriform and gelatinous fibers. Rays can be uniseriate and multiseriate, where prismatic crystals were commonly observed (Fig. 7).

### Sodium chloride concentrations

Cortex and stele of the saltwater cable root contain the highest concentration (583.70 ±  6.26 ppm), whereas saltwater wood showed the lowest concentration (64.75 ±  0.58 ppm) of sodium chloride. The average sodium chloride concentrations in all organs were significantly different between the saltwater and freshwater samples (*t*-test, *P* ≥ 0.034) (Table 2).

## Discussion

Anatomical features in the feeding root, anchor root, stem (secondary growth), and petiole of *Sonneratia caseolaris* from the saltwater and freshwater conditions are similar in most of their qualitative characters. However, some qualitative and the majority of quantitative characters showed the significant differences between saltwater and freshwater plants, particularly in their cable root, pneumatophore, stem (primary growth and wood) and leaf blade. The common characters are cortical aerenchyma, idioblasts with druse crystals, sclereids and mucilaginous cavity. The different characteristics include the distribution of tanniferous cells in certain tissues, the presence of gelatinous fibers in saltwater cable roots, leaf blade thickness, vessel density, and vessel diameter in the wood. The comparison of the anatomical characters between freshwater and saltwater plants are shown in the Table 3.

All types of *Sonneratia caseolaris* roots growing in saltwater appeared to develop periderm from a young stage. Periderm is a secondary tissue which is normally developed in secondary growth stage. Surprisingly in this case, periderm was observed even in young roots (with primary growth). This is accordance with observations in other mangrove species, including *Rhizophora* spp., *Ceriops* spp. (Thonglim, 2018), and *Bruguiera* spp. (Pornpromsirikul, 2017). Periderm is involved in not only a protection, but also infiltration of salt from soil and seawater (Kraisungnern, 1989). In this study, root periderm excluded 93.34% of sodium chloride from the saline water at 5,887 ppm. Reef & Lovelock (2015) reported similar levels of salt exclusion in other species by comparing the salt concentration in the soil (ranging from 6,000 to 16,000 ppm) with that in the xylem sap. The exclusion percentage ranged from 93.9% in *Avicennia marina* to 99.6% in *Bruguiera gymnorrhiza*. These results suggest that most of the mangrove species, including our study species, exclude most of the salt in the root tissue.

**Table 1:** Leaf blade thickness of *Sonneratia caseolaris* growing in saltwater and freshwater conditions. The values are displayed as mean ± standard deviation.

Leaf blade	Saltwater (µm)	Freshwater (µm)	*P*-value^*^
Adaxial epidermis	13.41 ±1.50	12.29 ±0.96	0.066
Palisade parenchyma on adaxial side	93.73 ±13.13	95.17 ±8.72	0.776
Spongy parenchyma	139.91 ±10.39	188.83 ±18.15	<0.001
Palisade parenchyma on abaxial side	74.99 ±7.51	82.34 ±6.69	0.033
Abaxial epidermis	12.83 ±1.91	10.20 ±0.95	0.002
Total thickness	334.86 ±15.72	388.83 ±19.07	<0.001

**Notes.**

* Note: *P*-value from *t*-test.

Flooding leads to an anaerobic environment that causes to respiration stress of mangrove plants. Roots of *S. caseolaris* in both conditions showed anatomical adaptations to cope with this problem. The submerged roots of this plant show a unique capacity of gas exchange by aerenchyma formation with abundant air spaces that allow oxygen transportation. Gas is taken through lenticels of pneumatophores which are aerial above the ground. Pneumatophores are connected to feeding roots and cable roots. Consequently, oxygen can be transferred to all parts of roots through aerating system (Armstrong, Brändle & Jackson, 1994; Purnobasuki & Suzuki, 2004; Purnobasuki & Suzuki, 2005). However, aerenchyma formation may reduce root functions such as water and mineral uptake and transportation (Moog, 1998).

Druse crystals are common in all organs of *S. caseolaris* and prismatic crystals presented in wood rays of plants from both studied environments*.* Crystal is a calcium oxalate compound which is derived from metabolic waste. Druse crystals can be observed accumulating along phloem of stem, petiole, and leaf blade. The vascular bundles were surrounded by calcium oxalate, because the precipitation of overload calcium will prevent the calcium from accumulating around the chlorenchyma cells which can affect cellular function. This adaptation is calcium regulation. In addition, other functions of crystals include plant protection, detoxification, ion balance, tissue support rigidity and even light gathering and reflection (Franceschi, 2001; Franceschi & Nakata, 2005).

**Table 2:** Sodium chloride concentrations in different tissues and organs of *Sonneratia caseolaris* growing in saltwater and freshwater environments. The values are shown as mean ± standard deviation.

		Saltwater (ppm)	Freshwater (ppm)	*P*-value^*^
Surrounding water		5885.00 ± 1.10	120.20 ± 0.98	<0.001
Cable root				
	Periderm	219.10 ± 5.46	268.04 ± 13.95	<0.001
	Cortex and stele	583.70 ± 6.26	229.56 ± 3.47	<0.001
Pneumatophore				
	Periderm	327.14 ± 4.12	168.22 ± 1.97	<0.001
	Cortex and stele	322.62 ± 1.55	220.74 ± 1.74	<0.001
Feeding root		388.12 ± 9.15	237.60 ± 2.89	<0.001
Anchor root		511.68 ± 6.40	265.74 ± 2.38	<0.001
Stem				
	Bark	201.00 ± 0.51	110.16 ± 1.29	<0.001
	Wood	64.75 ± 0.58	89.15 ± 0.57	<0.001
Leaf blade		229.28 ± 1.77	233.54 ± 3.06	0.034

**Notes.**

* Note: *P*-value from *t*-test.

**Table 3:** The comparisons of anatomical characters in each organ of *Sonneratia caseolaris* grown in saltwater and freshwater conditions. The values are shown as mean ± standard deviation.

Organs	Saltwater	Freshwater
Cable root	Gelatinous fiber (xylem)	No Gelatinous fiber
Pneumatophore	Tanniferous cell (cortex)	No Tanniferous cells
Stem (Primary growth)	Tanniferous cell (cortex)	No Tanniferous cells
Leaf blade	Tanniferous cell (palisade)	No Tanniferous cells
Vessel density	26.37 ± 4.37 pores/mm^2^	23.33 ± 2.11 pores/mm^2^
Vessel diameter	79.97 ± 10.51 µm	84.39 ± 12.46 µm

**Notes.**

* Note: *P*-value from *t*-test.

A number of astrosclereids were observed in all organs that contain aerenchyma. They serve as a mechanical support to increase strength in soft tissues. However, the sclereids are also present in leaf mesophyll. They may diminish leaf turgor (Naskar & Palit, 2015) or discourage herbivores (Kathiresan & Bingham, 2001).

Mucilaginous cells were found in adaxial palisade of leaves from both conditions. This character has been reported in other species of *Sonneratia*, and these cells may involve in reducing damages from wilting by water storing function (Gregory & Baas, 1989; Lyshede, 1977).

Tanniferous cells are present in most parts of the plants. However, the saltwater plants showed higher tannin than that of freshwater. These cells were observed in pneumatophore and palisade parenchyma of saltwater plants, but they were absent in freshwater plants, suggesting their role in coping with a high salinity condition. Tannin does play a role in iron elimination because the mangrove forest has a high concentration of iron (Defew, Mair & Guzman, 2005). It has an ability to chelate metal ions such as ferrous and interfere with the Fenton reaction and thereby retards oxidation (Amarowicz, 2007; Kimura & Wada, 1989). Anaerobic condition and sulphate soil lead to the occurrence of Fe toxicity in soil solution (Sperotto et al., 2010). A previous report found that iron overload in rice occurs in flooded soils when its roots take up high concentration of ferrous from soil to accumulate in leaf (Sahrawat, 2004). In this study, the tissues of *S. caseolaris* from saltwater deposits more tannins than those from freshwater, which suggests that the role of tannin may be trapping the excess iron. However, more detailed study on iron level in the soil and trapping mechanism will be needed to confirm the role of tanniferous cells.

The salinity is associated with leaf blade thickness of *S. caseolaris*. Leaf blade thickness is lower in the leaves from saltwater areas compared to the freshwater. It has been reported that salinity causes changing in mesophyll structure with decreasing intercellular spaces in leaf blade (Delfhine et al., 1998). In other words, the mesophyll thickness decreases in response to salinity due to the reduction in length of palisade and spongy parenchyma. Kodikara et al. (2017) reported that the percentage water content of seedling grown under the low salinity was significantly higher than high salinity condition. In contrast, Hoppe-Speer et al. (2011) and Sobrado (2007) reported that leaf blade thickness of *Rhizophora mucronata* and *Laguncularia racemosa* increased when salinity increased due to a higher water content in leaf blade. Most of the mangrove leaves are succulent due to development of a water storing hypodermis and strongly developed palisade parenchyma with small intercellular spaces to reduce salt concentration in the sap by transferring the salts into senescent leaves (Parida & Jha, 2010). In our study, leaf blade thickness of the saltwater plant was lower than that of the freshwater plant. This may be because *S. caseolaris* does not accumulate most of the salt in its leaf blades (Table 2), which led to the similar concentrations of salt in the leaves of freshwater and saltwater plants.

The average of vessel density of *S. caseolaris* was significantly higher in saltwater plants (26.37 pore/mm^2^) than those in freshwater plants (23.33 pore/mm^2^), which is similar to the observations for *Rhizophora mucronata* (Dissanayake et al., 2018; Schmitz et al., 2006) and *Laguncularia racemose* (Sobrado, 2007). A small diameter of vessels is known to decrease transport efficiency, but simultaneously provide greater hydraulic safety (Naskar & Palit, 2015). High salinity causes an osmotic stress and cavitation in vessels and subsequent embolism (Hacke & Sperry, 2001; Sperry & Tyree, 1988; Tyree & Sperry, 1989). The advantage of a higher vessel density is the increase of transport system via both pores (Mauseth & Plemons-Rodriguez, 1998; Villar-Salvador et al., 1997) and inter-vessel pits can be circumvented alternative routes when vessels become embolized (Tyree, Davis & Cochard, 1994).

Vessel diameters were significantly smaller in the saltwater plants (79.97 µm) than in the freshwater plants (84.39 µm). The wider vessels are advantageous for growing in freshwater because of the high transpiration rates, while, in saltwater condition, water uptake would be limited by a low soil water potential. Therefore, a narrow vessel reduces the risk of xylem embolisms that often occur in the mangrove environment (Dissanayake et al., 2018; Naskar & Palit, 2015; Schmitz et al., 2006; Sobrado, 2007).

Gelatinous fibers were observed in stem phloem and wood xylem of plants from saltwater and freshwater environment. These fibers accumulate less lignin, less pentosan and high cellulose contents, leading to the presence of an internal gelatinous layer in the lumen (Mellerowicz & Gorshkova, 2011). The important function is to protect the organ damage from the wind and storm surge (Yoshida et al., 2002). For *S. caseolaris* grown in the windy zone, the structures containing gelatinous fibers are necessary. Interestingly, these fibers were also found in cable root xylem of saltwater plants, but not found in freshwater plants. The cable roots of saltwater plants grow horizontal to the sea, leaving them exposed to tides and waves at all times. Therefore, the presence of gelatinous fibers can help protect these cable root from breakages in the saltwater environment.

All parts of the plants showed dramatically lower salt levels of sodium chloride than the environment in saltwater (Table 2). This is not true for freshwater plants. Since salt storage and salt secreting organs were not observed in *S. caseolaris*, this species uses the filtration at root as its main mechanism to eliminate excess salt. Based on our results, there are two ways of salt elimination in *S. caseolaris*: salt exclusion by periderm and salt trapping by tannin. Periderm is an insulating tissue which deposits suberin substance on cell wall. It provides an efficient barrier to water movement into root cells for exclude salt ion from saline water. In *Avicennia marina*, the suberization significantly limits passive ion and water transport to stele and blocks almost all apoplastic water (Moon et al., 1986). Sodium chloride concentrations in vacuole of living cells (cortex, pith) are higher than death cells (xylem). This implies that the salt ions were trapped in living cells. It was noted that the tissues with tanniferous cells showed low concentrations of sodium chloride.

Interestingly, some parts of the plants grown in freshwater deposited greater amount of salt than in the saltwater environment. Thus, it may be possible that this mangrove plant accumulates salt for growth and development. In *Bruguiera parviflora*, the highest growth and photosynthetic pigments are observed at 100 mM NaCl (Parida, Das & Mittra, 2004). However, the excess sodium chloride (400 mM and greater) decreases growth, photosynthetic pigments, protein, lipid, nitrate reductase activity and photosynthesis (Parida & Das, 2005).

We conclude that anatomical characters of *Sonneratia caseolaris* varied according to their salinity environment. While genetic differences could be responsible for the observed differences, the plasticity of the anatomical characters is more likely in our cases, because all of the “freshwater” *S. caseolaris* plants are transplanted out of the mangrove forest. No native freshwater plant is reported for this species. Therefore, local adaptation by different genotypes is unlikely. The different organs include cable root, pneumatophore, primary growth stem, leaf and wood. Most of salt from the environment is filtered by the peridermal tissue. Gelatinous fibers are present in stems and cable roots. Gelatinous fibers may play a role in reinforcement against strong wind, tides, and waves. The occurrence of tanniferous cells may relate salt elimination or iron elimination, as we observed a greater abundance of these cells in the saltwater samples. These within-species difference in anatomical structures between fresh and saltwater samples illustrate a unique set of adaptation that *S. caseolaris* employs to deal with the difficult environment of mangrove forests.

##  Supplemental Information

10.7717/peerj.10962/supp-1 Supplemental Information 1 Salt content measurements in ppm of *Sonneratia caseolaris*

10.7717/peerj.10962/supp-2 Supplemental Information 2 A transverse sections of four types of *Sonneratia caseolaris* root growing in freshwater A-C: cable root. D-E: pneumatophore. G-I: feeding root. J-L: anchor root. Abbreviations: ae, aerenchyma; pe, periderm.

10.7717/peerj.10962/supp-3 Supplemental Information 3 Leaf thickness measurements in ppm of *Sonneratia caseolaris*

## Additional Information and Declarations

### Field Study Permissions

The following information was supplied relating to field study approvals (i.e., approving body and any reference numbers): Samples of *S. caseolaris* were kindly supplied by the Sirinart Rajini Ecosystem Learning Center and authorized by the manager Komson Hongpadharakiree, in Prachuap Kiri Khan Province, next to the Gulf of Thailand.

### Data Availability

The following information was supplied regarding data availability: Raw measurements of salt contents and leaf thickness are available in Supplemental File.
