# Supplementary figures and images for: Comparative anatomy and salt management of Sonneratia caseolaris (L.) Engl. (Lythraceae) grown in saltwater and freshwater
Source: PeerJ. 2021 Feb 25;9:e10962. doi: 10.7717/peerj.10962 (PMC7916540; doi:10.7717/peerj.10962)

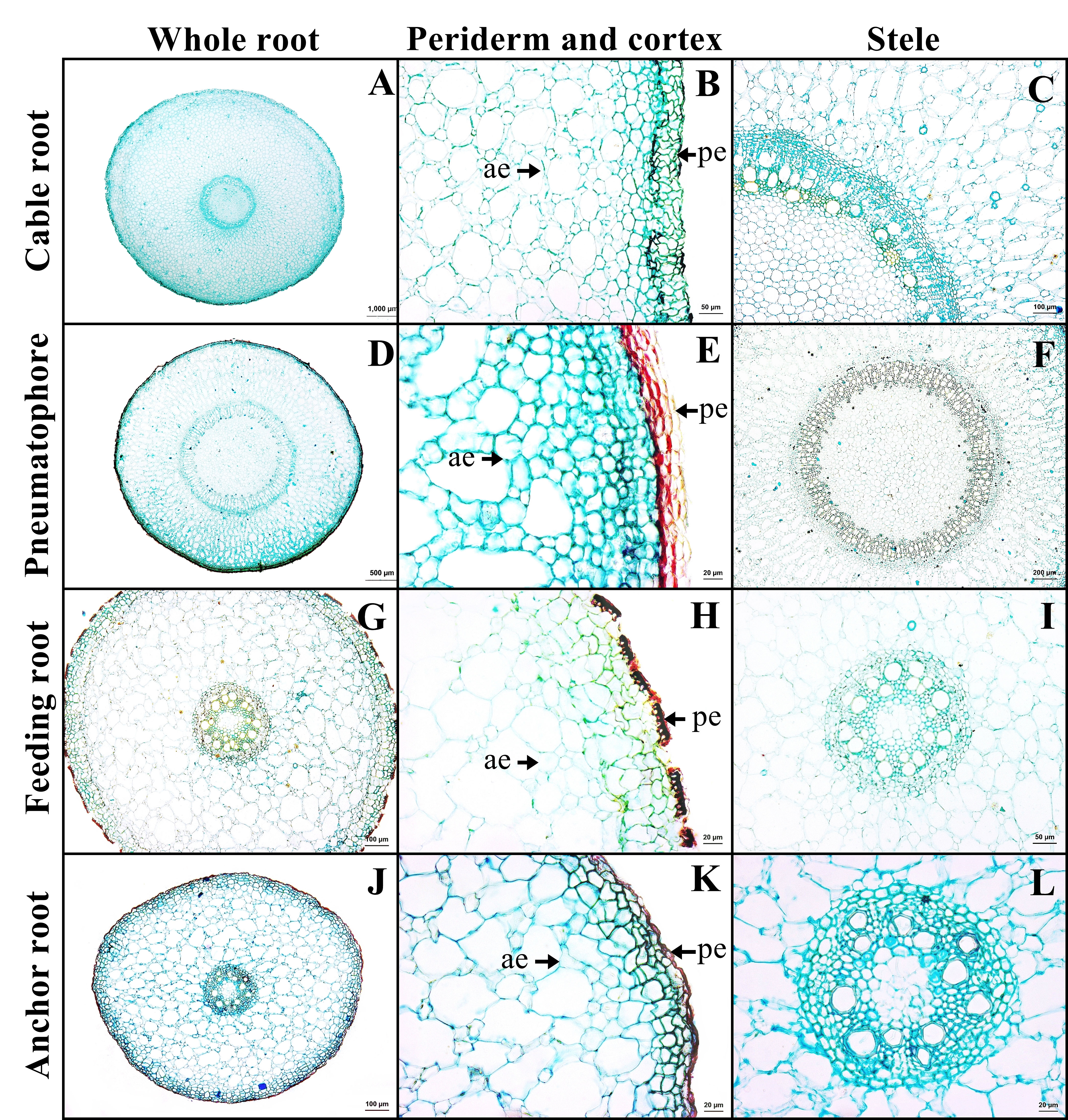

Supplement: Supplemental Information 2 — A-C: cable root. D-E: pneumatophore. G-I: feeding root. J-L: anchor root. Abbreviations: ae, aerenchyma; pe, periderm. [file peerj-09-10962-s002.jpg]
